# Molecular dynamics of the ERRγ ligand-binding domain bound with agonist and inverse agonist

**DOI:** 10.1371/journal.pone.0283364

**Published:** 2023-04-06

**Authors:** Santanu Sasidharan, Kamalakannan Radhakrishnan, Jun-Yeong Lee, Prakash Saudagar, Vijayakumar Gosu, Donghyun Shin

**Affiliations:** 1 Department of Biotechnology, National Institute of Technology, Warangal, Telangana, India; 2 Combinatorial Tumor Immunotherapy MRC, Chonnam National University Medical School, Hwasun-gun, Jeonnam, Republic of Korea; 3 School of Life Sciences, BK21 FOUR KNU Creative BioResearch Group, Kyungpook National University, Daegu, Republic of Korea; 4 Department of Animal Biotechnology, Jeonbuk National University, Jeonju, Republic of Korea; 5 Department of Agricultural Convergence Technology, Jeonbuk National University, Jeonju, Republic of Korea; Dr Reddy’s Institute of Life Sciences, INDIA

## Abstract

Estrogen-related receptor gamma (ERRγ), the latest member of the ERR family, does not have any known reported natural ligands. Although the crystal structures of the apo, agonist-bound, and inverse agonist-bound ligand-binding domain (LBD) of ERRγ have been solved previously, their dynamic behavior has not been studied. Hence, to explore the intrinsic dynamics of the apo and ligand-bound forms of ERRγ, we applied long-range molecular dynamics (MD) simulations to the crystal structures of the apo and ligand-bound forms of the LBD of ERRγ. Using the MD trajectories, we performed hydrogen bond and binding free energy analysis, which suggested that the agonist displayed more hydrogen bonds with ERRγ than the inverse agonist 4-OHT. However, the binding energy of 4-OHT was higher than that of the agonist GSK4716, indicating that hydrophobic interactions are crucial for the binding of the inverse agonist. From principal component analysis, we observed that the AF-2 helix conformation at the C-terminal domain was similar to the initial structures during simulations, indicating that the AF-2 helix conformation is crucial with respect to the agonist or inverse agonist for further functional activity of ERRγ. In addition, we performed residue network analysis to understand intramolecular signal transduction within the protein. The betweenness centrality suggested that few of the amino acids are important for residue signal transduction in apo and ligand-bound forms. The results from this study may assist in designing better therapeutic compounds against ERRγ associated diseases.

## Introduction

The nuclear receptor (NR) superfamily is a group of 48 ligand-regulated transcription factors in humans [1]. The amino acid sequence of the members of this superfamily has conserved regions, such as the DNA binding domain (DBD), which is highly conserved, the C-terminal ligand-binding domain (LBD), and the N-terminal domain, which is poorly conserved. The DNA-binding domain is responsible for the sequence-specific recognition of DNA in the regulatory regions of the target genes, while the C-terminal is capable of binding to small and lipophilic compounds that influence transcriptional regulation by interacting with cofactors. The LBD comprises 10–13 α-helices that are arranged like a three-layered sandwich, thereby regulating volume and function by specifically binding to molecules.

The estrogen-related receptor (ERR) subfamily consists of three orphan NRs, ERRα (NR3B1), ERRβ (NR3B2), and ERRγ (NR3B23), which are closely related to the estrogen receptors (ER) [2]. The regions of the DNA-binding domains of both classic ERs and orphan ERRs are conserved and therefore recognize common DNA-binding sites or response elements that are proximal to the target genes [3]. ERRs are capable of controlling classic ER target genes in the breast and bones [4, 5]. Although a certain degree of sequence identity exists between the ERR and ER LBD, there is a significant difference in their ligand binding capacities. ERRα and ERRβ were studied extensively before ERRγ was identified; therefore, not much is known about the role and dynamics of ERRγ [2, 6, 7].

ERRγ was first identified when it was linked to a region critical for Usher syndrome type IIa. Later, the receptor was discovered to interact functionally with the NR co-activator glucocorticoid receptor-interacting protein (GRIP) [3, 8]. This protein receptor is primarily expressed in metabolic tissues such as muscle, liver, brain, heart, and adipose tissues. The complete role and function of the receptor have not yet been elucidated, as ERRγ-null mice were found to be non-viable after birth [9]. However, tissue-specific ERRγ knock-out mice, ERRγ-specific ligands that can modulate the transcriptional activity of the receptor, and studies involving gain/loss of function have allowed researchers to understand the functions of this unique receptor [10, 11]. Indeed, studies have shown that ERRγ plays a vital role in the metabolic functions of the liver, such as the regulation of glucose, alcohol, and lipids, along with iron metabolism and modulation of specific gene expression in endocrine and metabolic processes [12, 13]. In addition, it is now well known that abnormal regulation of ERRγ results in hepatocellular carcinoma, as it regulates the expression of microRNA and DNA methyltransferase [14, 15].

**Fig 1:**
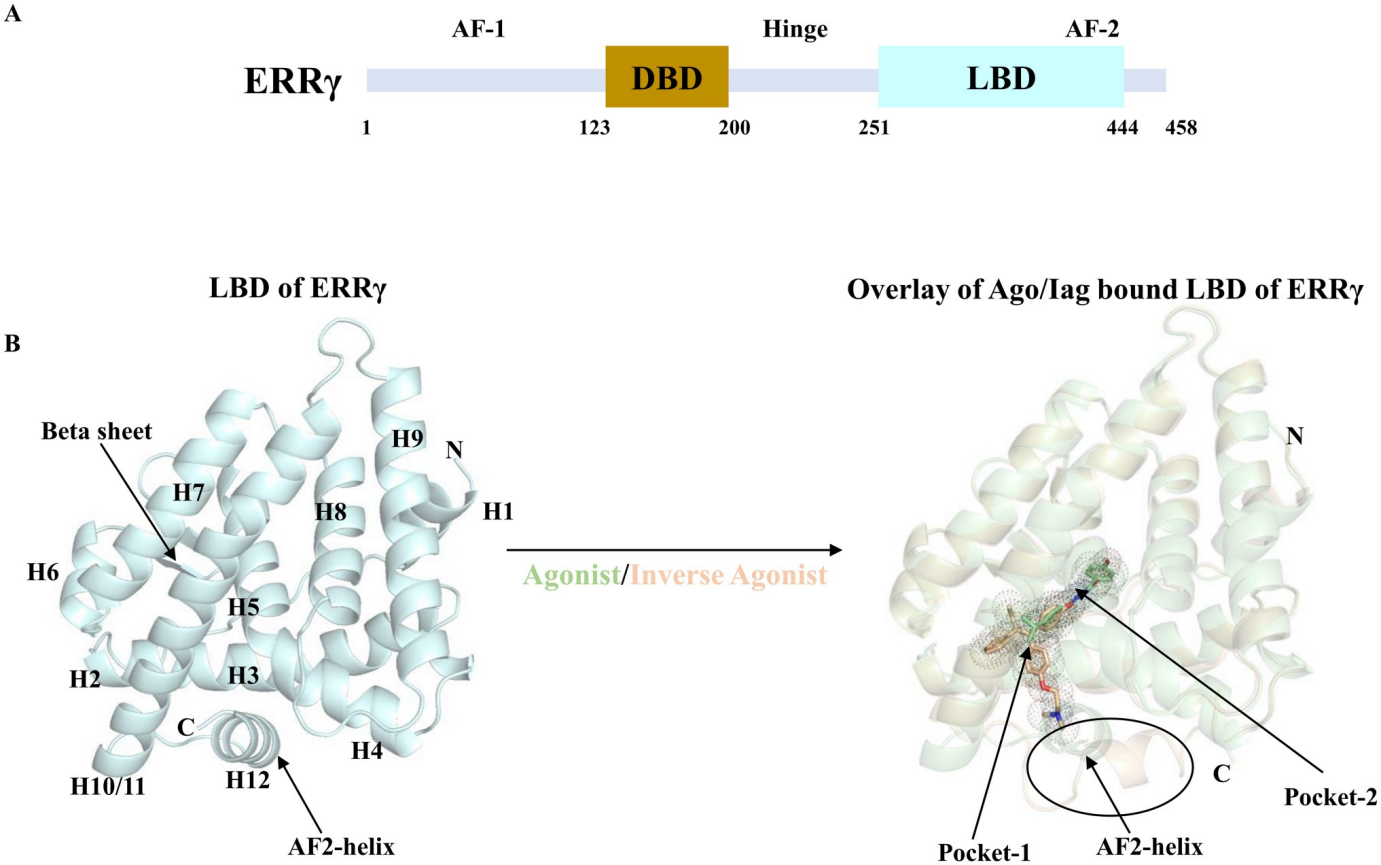
Domain organization and structure of ERRγ **(A)** The full-length domain organization of ERR**γ. (B)** Ligand-binding domain (LBD) structure of ERR**γ** which comprises of 12 helices and a β-sheet (left). Agonist- (pale green color) and inverse agonist- (wheat color) bound ERR**γ** structures are overlaid (right). Ligands are shown in stick representation with their respective pockets shown in dots. AF2-helix conformational differences are shown in the black circle.

As discussed earlier, ERRγ exhibits transcriptional activation of target genes in the absence of specific regulatory ligands; hence, the receptor is assigned as a constitutively active orphan NR. Structurally, ERRγ houses an N-terminal activation function (AF-1) domain, a central zinc finger DNA-binding domain (DBD) that binds to monomers or dimers to the core sequence TCAAGGTCA, referred to as the ERR response element (ERRE), and a C-terminal AF-2 domain that interacts with co-regulators (Fig 1). The crystal structure of the inverse agonist shows that the binding of the compounds to the small ligand pocket results in the displacement of the AF-2 helix and consecutively, the binding of the coactivator is blocked [16]. On the other hand, its agonist GSK4716, a micromolar acyl hydrazone ERRγ agonist, is capable of binding and activating receptors similar to PGC-1α, [17] but is 50 times more selective towards ERRγ. The X-ray crystal structure of the apo receptor has been shown to have an LBD in the active conformation with the AF-2 helix, which allows the binding of the coactivator [18]. The ligand-binding pocket of ERRγ has a total volume of 280 Å^3^, which is extremely small compared to that of ERα, which has a volume of 370 Å^3^ [16, 19]. To date, there are no endogenous small molecule ligands that have been discovered for ERRγ [20]. However, fasting-induced co-activator protein peroxisome proliferator-activated receptor γ coactivator 1-α (PGC-1α) has been known to bind to AF-2 and activate the receptor [21–23]. In addition to PGC-1α, GRIP-1, a co-activator, and co-repressors such as small heterodimer partner (SHP) and small heterodimer partner-interacting leucine zipper protein (SMILE), bind to AF-2 for functional activity [3, 24–26]. In addition to small proteins, studies have found that high-affinity ER inhibitors such as diethylstilbestrol [3] and 9-hydroxytamoxifen (4-OHT) [4] also have the capacity to be inverse agonists of ERRγ.

In the current study, we performed all-atom molecular dynamics simulations to identify the intrinsic dynamics associated with the binding of the inverse agonist 4-OHT and the agonist GSK4716 to ERRγ. Previous studies have shown the effective use of molecular dynamics simulations to understand molecular interactions, allostery [27] and intrinsic dynamics of macromolecule complexes [28–32]. By utilizing the apo structure (ERRγ^apo^), GSK4716-bound ERRγ (ERRγ^ag^), and 4-OHT-bound ERRγ (ERRγ^iag^), the binding free energy between the ERRγ and ligand was studied. In addition, conformational changes with respect to the LBD and AF-2 helix were also analyzed. We further report the key amino acid residues from the betweenness centrality analysis, which may be important for the functional role of ERRγ.

## Materials and methods

### Computational resource and analysis of data

All computational works were performed on a Linux (Ubuntu) workstation. All-atom molecular dynamics (MD) simulations were performed using GROMACS v5.1.4 [33, 34] and the trajectories were analyzed using the in-built modules of the software. The residue network analysis was conducted using the NAPS web server [35, 36]. All graphical images were generated using PyMol [37]. Graphs were plotted using Microsoft Excel, and free-energy landscape (FEL) plots were generated using Mathematica.

### Structure preparation of apo and ligand bound ERRγ

Previously, several structures have been solved for the ERRγ binding with agonist/ inverse agonist as well as coactivator molecules [16]. Since the aim of the study is to identify the crucial residues as well as binding free energies of agonist and inverse agonist, we considered only the ERRγ^apo^ and ERRγ bound with agonist or inverse agonist. At first, the crystal structures of ERRγ^apo^, ERRγ^ag^, and ERRγ^iag^ were obtained from the Protein Data Bank (PDB) [16]. For ERRγ^apo^, we retrieved the crystal structure of ERRγ ligand binding domain (PDB id: 2GP7) with a resolution of 2.45 Å. Whereas, for ERRγ^ag^, and ERRγ^iag^, the X-ray crystal structure of ERRγ ligand binding domain with agonist (GSK4716) and inverse agonist (4-OHT) (PDB id: 2GPP and 2GPU, respectively) were retrieved with a resolution of 2.6 Å and 1.7Å, respectively. Water molecules and ions were removed from all the three structures. In ERRγ^ag^, the RIP140 protein bound to the crystal structure was removed, and the structure was saved using only GSK4716 as the ligand. The side chains of few residues in ERRγ^apo^ and ERRγ^iag^ had more than one conformation as well as missing in ERRγ^ag^ as a result of low resolution from X-ray scattering in that region. Therefore, we applied mutation with the same residue to acquire better sidechain conformation with probable rotamers using mutation option in PyMol.

### All-atom molecular dynamic (MD) simulation

Once the structures were prepared, the three systems were subjected to MD simulations using GROMACS v5.1.4 with the AMBER-ff99SB-ILDN force field [38]. Topology files of the Amber force field for the ligands were generated using the ACPYPE web server [39]. Some of the mismatching atom types of the corresponding atoms from the ACPYPE server were renamed to be compatible with the corresponding GROMACS atom types. Initially, the structures were placed in a dodecahedron box with a 1.2 nm distance between the protein and box wall. All three systems were solvated using the TIP3P water model and then neutralized by replacing the solvent molecules with either Na^+^ or Cl^-^ ions. The systems were energy-minimized by the steepest descent algorithm with a maximum energy tolerance of 1,000 kJ/mol. The systems were then equilibrated using NVT and NPT ensembles for periods of 100 and 500 ps, respectively. Modified Berendsen thermostat (v-rescale) [40] was employed at 300 K for the NVT ensemble, while we employed Parrinello-Rahman barostat at 1 bar for NPT equilibration [41]. Following equilibration, the production run was performed for 500 ns, without considering any restraints. The long-range interactions were maintained with the help of the Particle Mesh Ewald algorithm, while the cut-off for short-range and long-range electrostatic interactions was set to 1.0 nm. The simulation grid space was set at 0.16 nm for FTT with fourth-order cubic interpolations. The simulations were performed with a time-step of 2 fs, and 2 ps coordinates were collected for the entire simulation trajectory. Subsequently, the trajectories were analyzed using in-built trajectory tools. The analysis (RMSD, Rg, SASA and H-bonds) were performed using 5000 frames (with 100 ps snapshot) from the entire trajectory. Binding free energy calculations were performed using the gmx mmpbsa tool (Molecular Mechanics Poisson-Boltzmann Surface Area) using the stable trajectory of 100ns (240 to 340 ns) of RMSD with 100 ps coordinates (1000 frames) [42].

### Principal Component Analysis (PCA)

PCA can be employed to understand the dominant and large-scale motions of the three systems along with their collective modes. Initially, a covariance matrix was constructed for the three systems based on the fluctuations of the protein [43]. The coordinates for all three systems were extracted every 100 ps from the last 450 ns, the translational and rotational motions were eliminated, and the covariance matrix was diagonalized. After the sum of the eigenvalues for ERRγ^apo^, ERRγ^ag^, and ERRγ^iag^ was calculated, the gmx anaeig was used to analyze the eigenvectors [44, 45]. The free energy landscapes (FEL) were constructed using the gmx sham tool to obtain the minimum-energy configurations and metastable states.

### Residue interaction network (RIN) and Betweenness centrality (C_B_)

To further understand the intramolecular signal transduction within the protein, a non-weighted residue-residue interaction network was constructed using the NAPS web server [36] for the low-energy structures of apo and ligand-bound forms of ERRγ. In the network, each amino acid residue represents a node and the contacts represent edges. For network construction, we used a Cα-Cα pair maximum threshold of 7 Å with a non-weighted edge. The effect of the side chains, that is, the Cα backbone and the heavy atoms that are far away from the Cα of the side chains were considered by taking two coarse grain centers per residue. Contact was assumed to be established only if the above-defined atoms between two residues in the protein were present at a distance of less than 7 Å [46]. Using the residue interaction network, we calculated the betweenness centrality (C_B_). C_B_(u) measures signal transduction within the protein and is used to identify the central node in a protein.

$$C_{B}\left(u\right)=\sum_{s\neq u\epsilon V}\sum_{t\neq u\epsilon V}\sigma_{st}(u)/\sigma_{st}$$

where σ_st_ (u) is the shortest path that connects nodes s and t through node u, and σ_st_ is the shortest path that connects nodes s and t [35].

## Results

### Structural deviation and compactness of both apo and ligand-bound ERRγ

**Fig 2:**
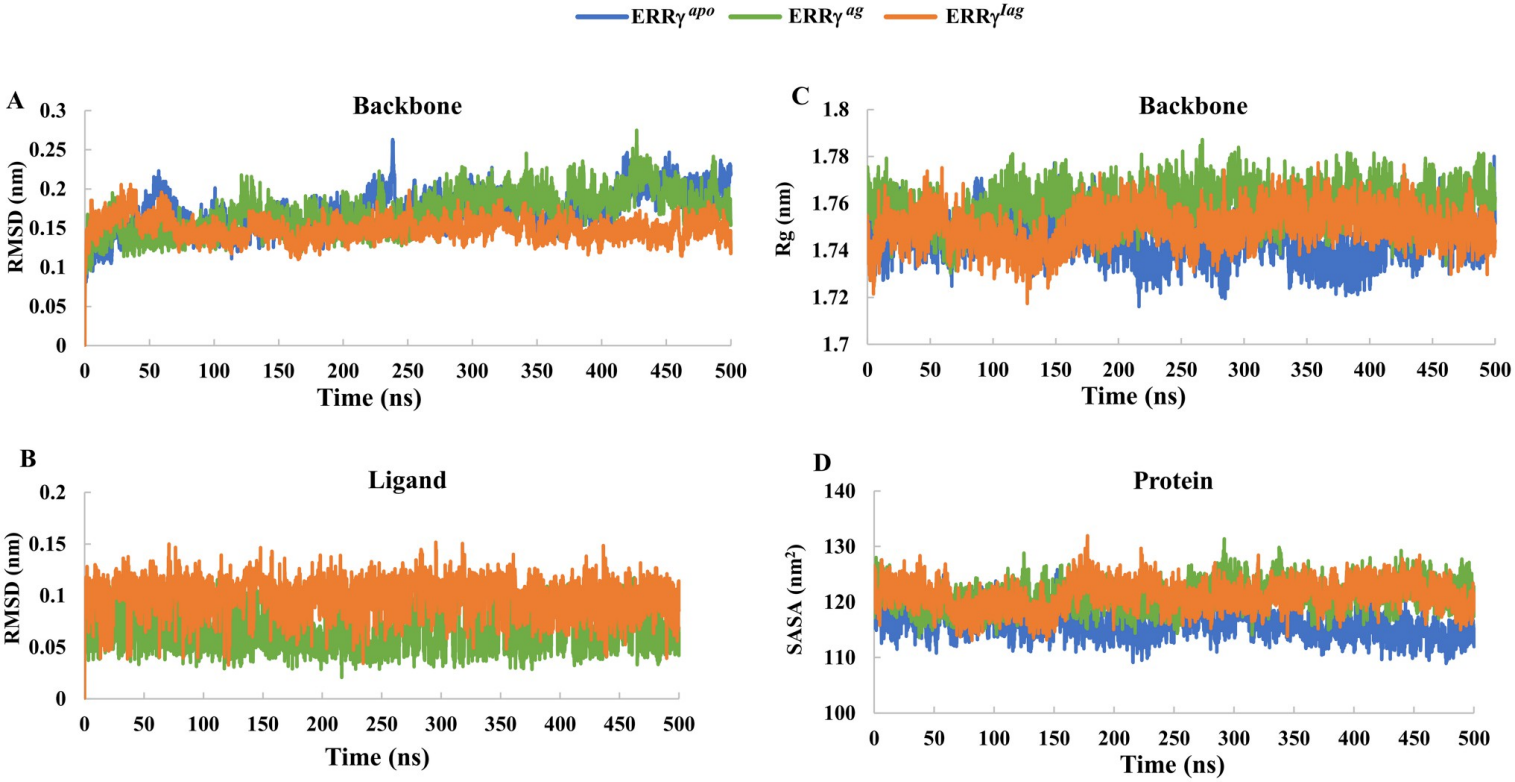
Trajectory analysis of ERRγ^apo^, ERRγ^ag^ and ERRγ^iag^: **(A)** RMSD of ERRγ^apo^, ERRγ^ag^ and ERRγ^iag^ backbone atoms. **(B)** RMSD of agonist and inverse agonist **(C)** Radius of gyration (Rg) of ERRγ^apo^, ERRγ^ag^ and ERRγ^iag^ backbone atoms **(D)** Solvent accessible surface area (SASA) of ERRγ^apo^, ERRγ^ag^ and ERRγ^iag^.

The ERRγ^apo^ and ligand-bound forms (ERRγ^ag^ and ERRγ^iag^) were analyzed for conformational deviations with respect to the initial structure during simulations. The root mean square deviation (RMSD) of backbone atoms of all three models was calculated and the measured average was 0.1724 ± 0.02 nm, 0.1691 ± 0.02 nm and 0.1489 ± 0.01 nm for ERRγ^apo^, ERRγ^ag^ and ERRγ^iag^, respectively. As shown in Fig 2A, all three systems showed similar structural deviations during the simulations indicating that the systems were well equilibrated. In particular, ERRγ^iag^ was largely stable compared to ERRγ^apo^ and ERRγ^ag^. When the RMSD of the ligands was analyzed, the agonist and inverse agonist showed lesser deviations from the initial structure, indicating that they were stable inside the ERRγ-LBD binding pocket (Fig 2B). Furthermore, the compactness of the protein was measured by calculating the radius of gyration (Rg) of the three systems over the simulation period. ERRγ^apo^, ERRγ^ag^ and ERRγ^iag^ had relatively stable compactness for ligand bound forms compared to ERRγ^apo^ suggesting a structural alteration upon ligand binding (Fig 2C). Taken altogether from the RMSD and Rg of the models, we assume that the three models exhibited fewer fluctuations and only marginal changes in the values, which suggests that there might not be large conformational changes upon ligand binding to ERRγ compared to ERRγ^apo^, except for few local structural rearrangements [16]. To further corroborate the conformational stability of the apo and complex models, we measured the solvent-accessible surface area (SASA). The SASA values of ERRγ^apo^ ranged from 108 to 125 nm^2^, whereas those of ERRγ^ag^ and ERRγ^iag^ ranged between 113–131 nm^2^ (Fig 2D). The smaller SASA values of apo ERRγ compared to the ligand-bound forms suggest that ligand binding induced small structural alterations in ERRγ, which might have caused an increase in the SASA of the ligand-bound forms of ERRγ.

### Residue flexibility of both apo and ligand bound ERRγ

**Fig 3:**
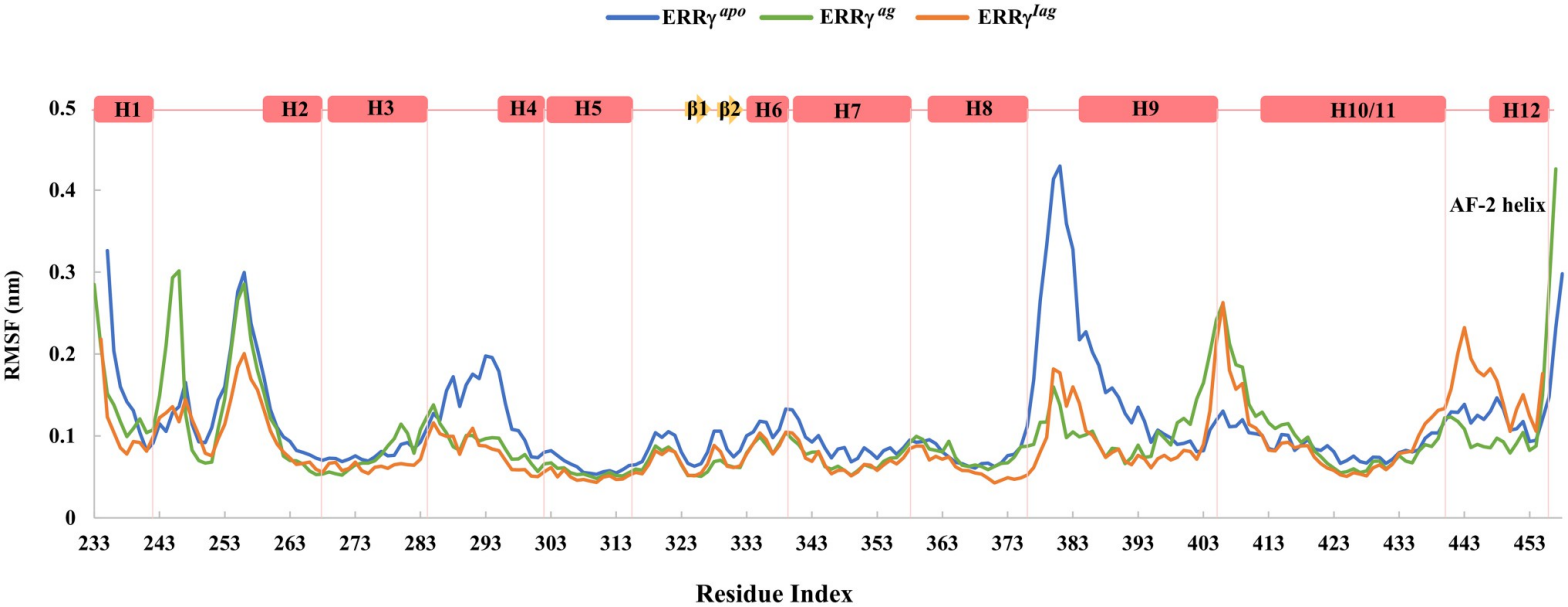
Root mean square fluctuation (RMSF) of ERRγ^apo^, ERRγ^ag^ and ERRγ^iag^: RMSF of the backbone atoms of ERRγ^apo^, ERRγ^ag^ and ERRγ^iag^.

To understand the intrinsic dynamics of ERRγ^apo^, ERRγ^ag^, and ERRγ^iag^ upon binding to the ligands, we performed a root mean square fluctuation (RMSF) analysis for backbone atoms. The results are presented in Fig 3, where ERRγ^apo^ exhibited large fluctuations only in the loop regions of the simulated LBD structure, particularly, the loops connecting helix 3 to helix 4 and helix 8 to helix 9. When we compared the RMSF of ERRγ^ag^ to that of ERRγ^apo^, we observed high residue fluctuations for ERRγ^ag^ in the loop region of 243–248, suggesting that the region is crucial for agonist binding as reported in the crystal structure [16]. Other loop regions were largely stable for ERRγ^ag^ indicating that the binding of the agonist GSK4716 to ERRγ caused rigidification of the loop regions, i.e., residues 285–300 and 375–395 compared to ERRγ^apo^ (Fig 3). On the other hand, when the RMSF of ERRγ^iag^ was compared to ERRγ^apo^, a trend similar to that of ERRγ^ag^ was observed in the loop regions, except in the region of the AF-2 helix (the loop connecting helix 12). The results strongly suggests that the displacement of the AF-2 helix conformation was necessary to block the functional activity of ERRγ upon inverse agonist binding [16]. It is worth mentioning that both ligand-bound forms of ERRγ exhibited an overall reduction in fluctuations, indicating that both ligands stabilize ERRγ structure upon binding (Fig 3).

### Hydrogen bonds and binding free energy of ligand bound ERRγ

**Fig 4:**
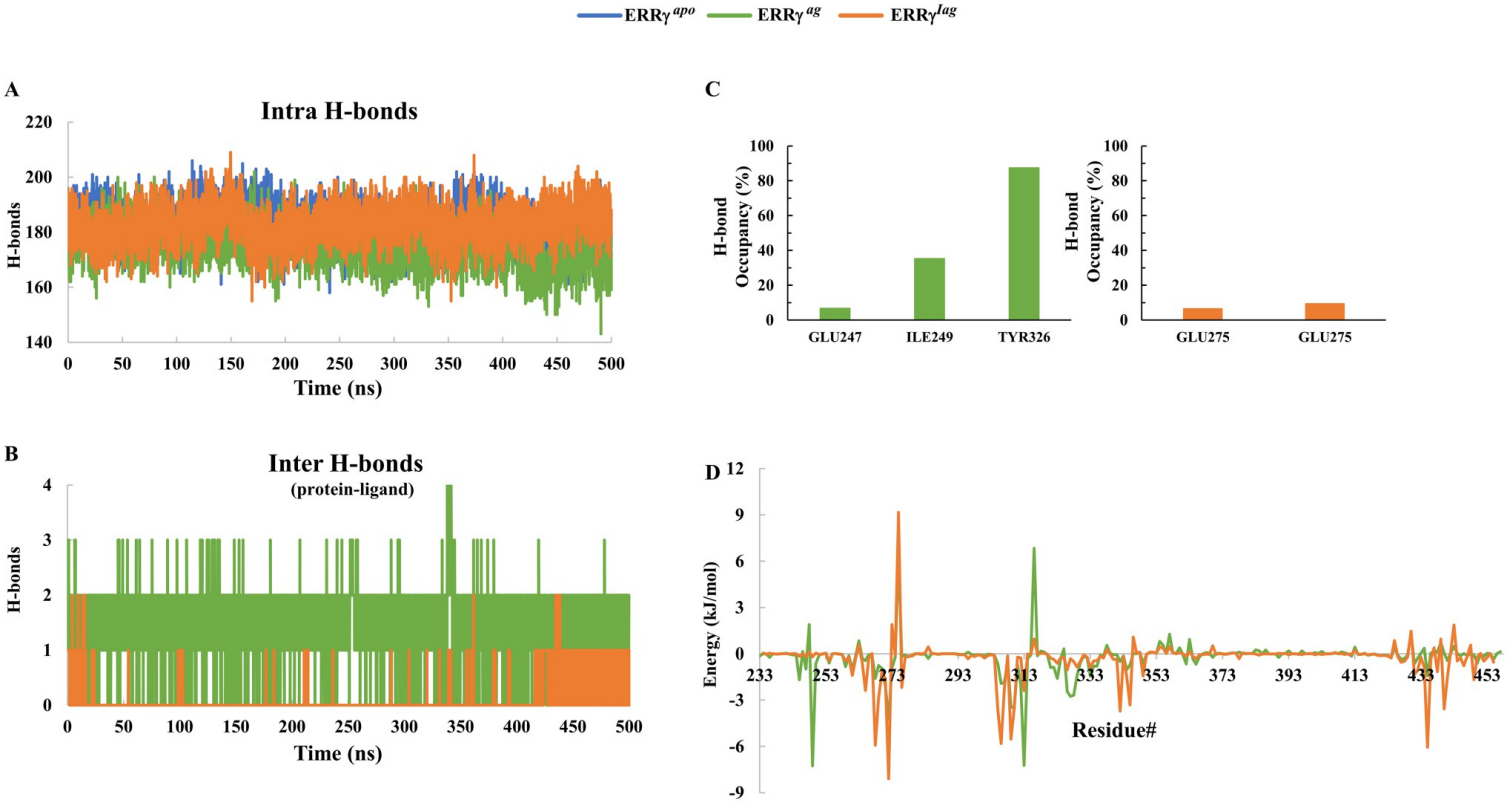
H-bond and free energy decomposition of ERRγ^apo^, ERRγ^ag^ and ERRγ^iag^ complex: **(A)** Number of intra H-bonds in ERRγ^apo^, ERRγ^ag^ and ERRγ^iag^. **(B)** H-bonds formed between agonist/inverse agonist and ERRγ. **(C)** The percentage of H-bond occupancy between the key residues with the ligands. The two bars of E275 in ERRγ^iag^ represent two atoms that act as either an acceptor or donor. **(D)** Energy decomposition of the residues in ERRγ^ag^ and ERRγ^iag^.

The intra H-bonds were calculated for the trajectories of the three systems (i.e., ≤ 3.5 Å distance and ≤ 30° angle). ERRγ^apo^ had a range of 158 to 205 H-bonds, with an average of 182 ± 7 bonds. ERRγ^ag^ had an average of 175 ± 7 intra H-bonds, whereas ERRγ^iag^ had 182 ± 7 H-bonds (Fig 4A). The intra H-bonds of ERRγ^ag^ and ERRγ^iag^ ranged between 143–202 and 155–209, respectively. We further examined the hydrogen bonding between the ligands and ERRγ structure. The number of inter H-bonds in ERRγ^ag^ ranged between 0–4 bonds while those in ERRγ^iag^ ranged between 0–2 bonds (Fig 4B). The LBD of ERRγ consists of two pockets, pockets 1 and 2. From the crystal structures obtained from PDB, the agonist GSK4716 bound to pocket 2 with a volume of 390 Å^3^ while 4-OHT bound to pocket 1 with a volume of 280 Å^3^ [16]. Therefore, the binding of inverse agonist 4-OHT to pocket one has a lower H-bond but causes the AF-2 helix to displace. In addition, we assessed the H-bond occupancy during simulations, which suggested that I249 and Y326 were key residues with occupancy of ~35% and ~85% in ERRγ^ag^, whereas, for ERRγ^iag^, we observed only E275 (acts as both acceptor and donor) with occupancy of ~6%. (Fig 4C).

We further calculated the binding free energies of the agonist and the inverse agonist of ERRγ. The binding free energy of the inverse agonist was higher than that of the agonist bound to ERRγ. From the individual energy contributions, the van der Waals energy and non-polar energy contribute significantly to the higher binding energy of inverse agonists to ERRγ (Table 1), showing that hydrophobic interactions are crucial for inverse agonist binding compared to agonists. We performed energy decomposition studies to gain a better understanding of the residues contributing to the energies. Residues E245, K248, I249, D261, L268, L271, A272, E275, M306, E307, L309, I310, G312, V313, R316, E323, Y326, A327, D328, D329, D333, D344, K357, K430 and F435 has significant energy changes while for inverse agonist, energy changes were observed for D261, L265, L268, C269, D270, L271, A272, D273, E275, L276, W305, M306, E307, L309, I310, V313, R316, Y326, L342, D344, L345, N346, I349, K430, A431, V432, F435, I438, L440, K443 and L449 (Fig 4D).

**Table 1:** Binding free energy calculations of ERRγ^ag^ and ERRγ^iag^.

Components	ERRγ^ag^	ERRγ^iag^
E_vdw_ (kJ/mol)	-201.68 ± 10.08	-241.83 ± 11.19
E_elec_ (kJ/mol)	-39.61 ± 11.5	-20.4 ± 5.45
G_polar_ (kJ/mol)	141.16 ± 13.6	130.9 ± 11.92
G_non-polar_ (kJ/mol)	-18.59 ± 0.7	-23.71 ± 0.81
ΔG_bind_ (kJ/mol)	**-118.72 ± 12.97**	**-155.06 ± 12.61**

**Fig 5:**
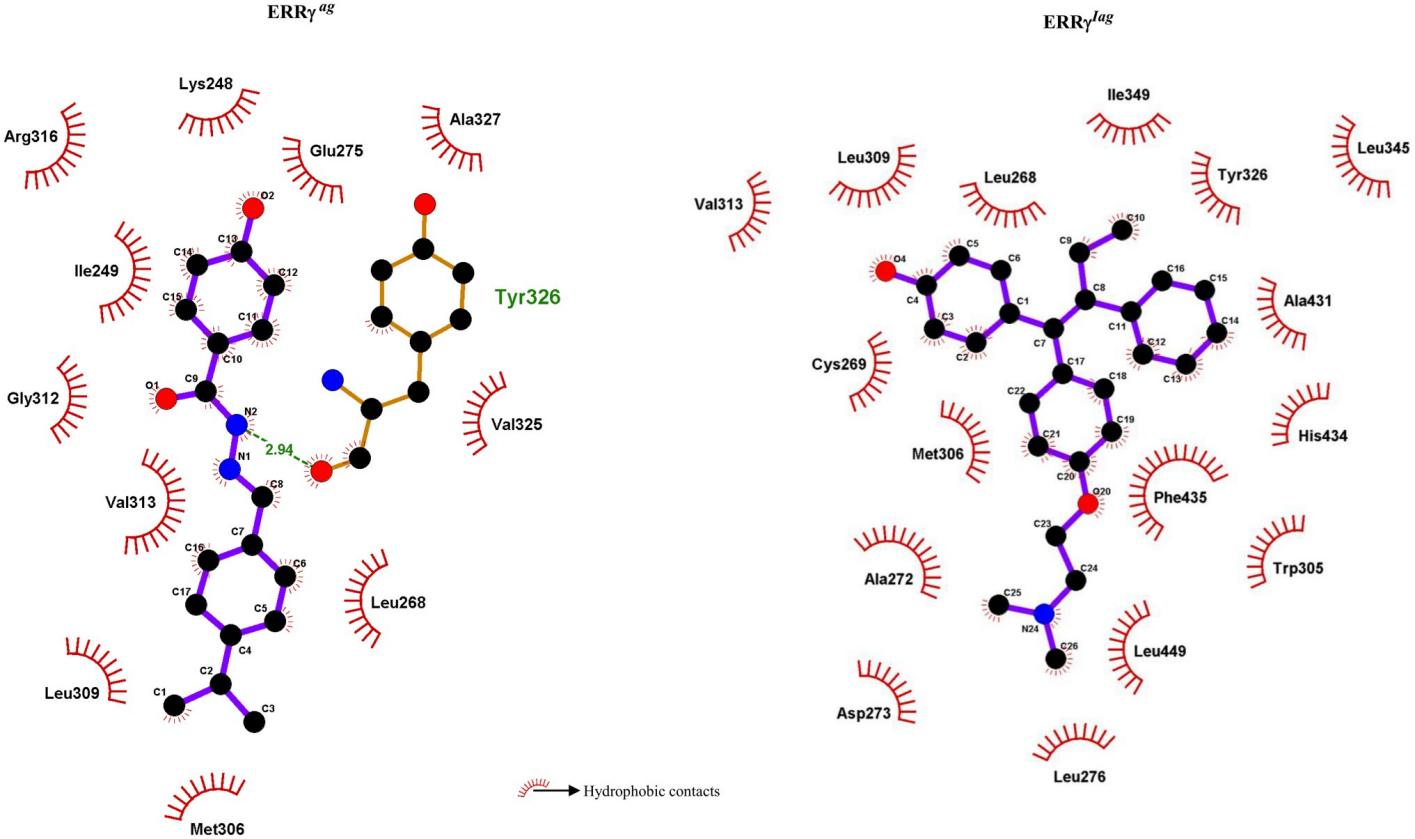
Interaction analysis: The 2D interaction pattern between agonist/inverse agonist and ERRγ.

To check the interactions between ligands and ERRγ, we extracted low-energy structures for both agonist and inverse agonist-bound forms. For ERRγ^ag^, the ligand formed hydrogen bonds with Y326 after simulation, whereas, for inverse agonist, no hydrogen bonds were observed. However, we observed more hydrophobic contacts for inverse agonist compared to agonist corroborating the binding free energy calculations (Table 2 and Fig 5).

**Table 2:** Comparison of interaction between the ligands and ERRγ structure.

Structure	Interactions	Before simulations	After simulations
ERRγ^ag^	H-bonds	Y326 and D328	Y326
	Hydrophobic Contacts	K248, I249, L268, M306, L309, G312, V313, R316, V325, A327 and F435	K248, I249, L268, E275, M306, L309, V313, G312, R316, V325 and A327
ERRγ^iag^	H-bonds	E275, R316	Nil
	Hydrophobic contacts	L265, L268, C269, A272, D273, W305, L309, Y326, L342, A431, H434, F435, and L440	L268, C269, A272, D273, L276, W305, M306, L309, V313, Y326, L345, I349, A431, H434, F435, and L449

In order to analyze the mutational impact of the residues involved in hydrophobic interaction with ligands, we performed in-silico mutagenesis for a few hydrophobic residues common for both agonist and inverse agonist bound ERRγ after simulations (i.e., L268, M306, L309 and V313) (Table 2) and the residues important for AF2-helix displacement in ERRγ^iag^ (F435 and F450). We replaced the amino acids (hydrophobic to hydrophobic or aromatic to aromatic), e.g. L309 to V309, to check whether like-to-like amino acid mutations could maintain their role in ligand binding and protein stability. The protein design module from the molecular operating environment (Chemical Computing Group, Montreal, Canada) was used to calculate the relative binding affinity (dAffinity) and thermal stability (dStability) of the mutated ERRγ structures (Table 3) as reported in previous studies [47, 48]. A positive dAffinity and dStability value suggest lower binding affinity and reduced stability of the mutants, respectively. The observed dAffinity values for the mutations of both ERRγ^ag^ and ERRγ^iag^ complex showed that like-to-like mutations may not largely affect the binding affinity or stability of the protein. However, the positive dAffinity and dStability values observed for M306, and L309 strongly reflect the importance of these residues for ligand binding and stability of ERRγ.

**Table 3:** Computation mutagenesis of hydrophobic residues involved in ligand binding. dAffinity and dStability represent the relative binding affinity and relative thermostability of the mutated to wild-type ERRγ, respectively.

Mutations	ERRγ^ag^		ERRγ^iag^	
	dAffinity	dStability	dAffinity	dStability
L268V	-0.0589	1.3912	-0.2264	1.5318
M306C	0.1117	2.0693	0.4198	2.4263
L309V	0.0537	1.3796	0.1108	1.4037
V313L	-0.2469	0.7286	-0.0231	0.6202
F435Y	-0.2486	0.5015	-0.1490	0.8078
F450Y	-0.0324	0.9927	-0.0470	1.1926

### Principal components (PC) and Free energy landscape (FEL) of both apo and ligand-bound ERRγ

**Fig 6:**
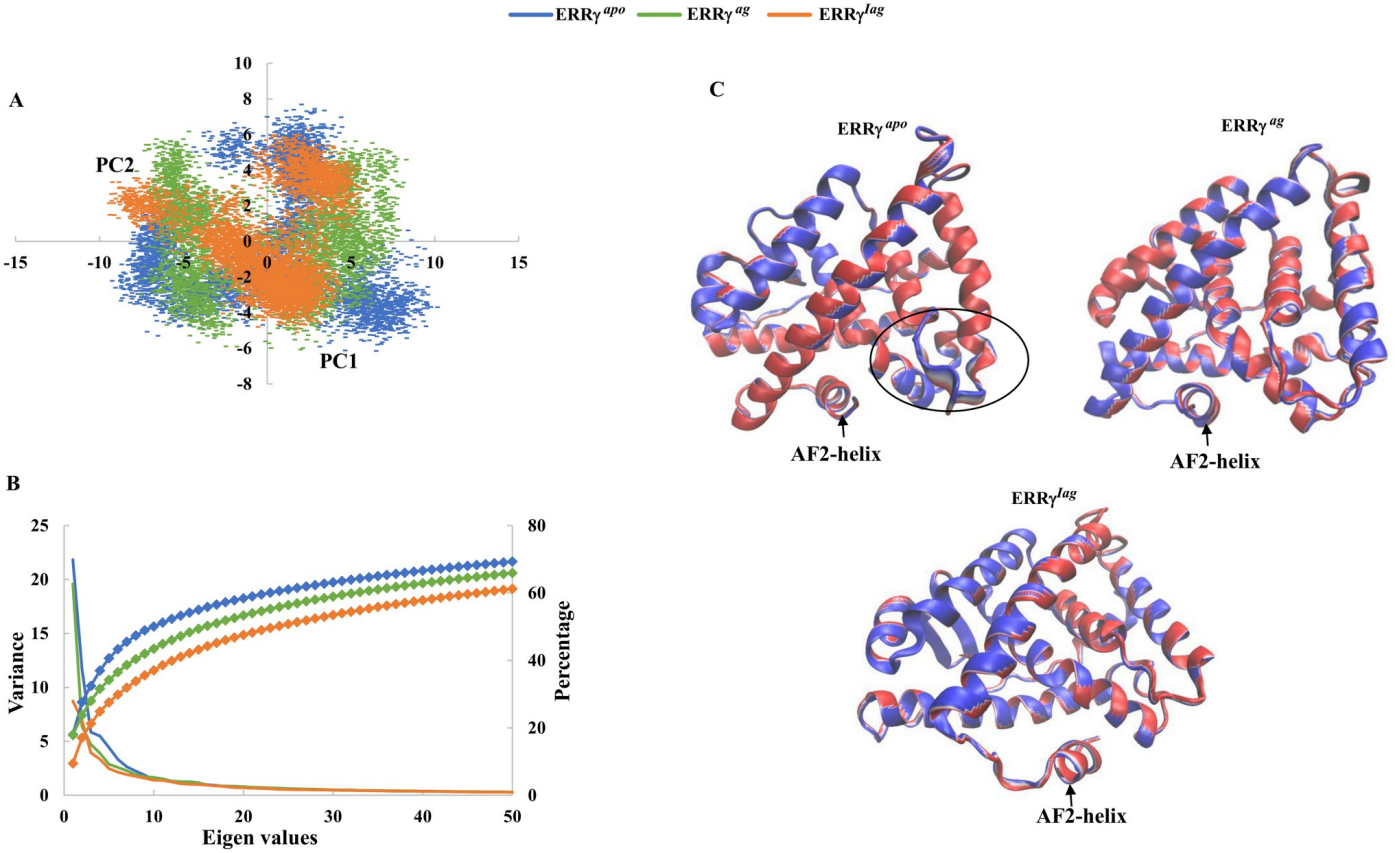
Principal component analysis of ERRγ^apo^, ERRγ^ag^ and ERRγ^iag^: **(A)** Principal component analysis of ERRγ^apo^, ERRγ^ag^ and ERRγ^iag^ projected by PC1 and PC2 onto the phase space. **(B)** The first 50 eigenvectors, corresponding to the best eigenvalue principal components, are represented in the plot along with their associated cumulative fluctuations. **(C)** The conformational changes, minimum (red) to maximum (blue) observed for ERRγ^apo^, ERRγ^ag^, and ERRγ^iag^ using PC1 and PC2 with 30 conformations extracted using the extreme module. The black circle represents the large conformational changes in the loop between helix-8 and helix-9 in ERRγ^apo^.

To understand the distribution of large motions responsible for the functional dynamics of the proteins, we performed a principal component analysis. The eigenvalues for the projections were calculated, and the projections with the largest eigenvalues, PC1 and PC2, were plotted (Fig 6A). The spread of the distribution of points, where each point represents a conformation of the protein, will be greater if the conformational changes in the protein are large. As shown in Fig 6A, the spread of PC1 and PC2 of ERRγ^apo^ was larger than those of ERRγ^ag^ and ERRγ^iag^. This is possibly due to the absence of ligands, which allowed conformational freedom in ERRγ^apo^. When ERRγ^ag^ and ERRγ^iag^ were compared, ERRγ^ag^ showed a slightly larger spread. The cumulative percentages of motion of the first 50 eigenvectors of ERRγ^apo^, ERRγ^ag^, and ERRγ^iag^ were 69.3%, 65.9% and 61.2%, respectively (Fig 6B). This is also in line with the large fluctuations in the residues recorded in ERRγ^apo^ and ERRγ^ag^. The results suggested that the binding of ligands to ERRγ allowed ERRγ^ag^ and ERRγ^iag^ to be highly stable in the trajectory analysis. We further extracted the extreme conformations for PC1 and PC2 to understand the regions that exhibited large conformational changes in all three systems. As expected, large conformational changes for apo ERRγ in the loop were seen between helix-8 and helix-9 similar to the RMSF results; however, no large structural variations were observed in the ligand-bound forms, strongly indicating that ligand binding may stabilize the whole structure compared to apo ERRγ (Fig 6C).

**Fig 7:**
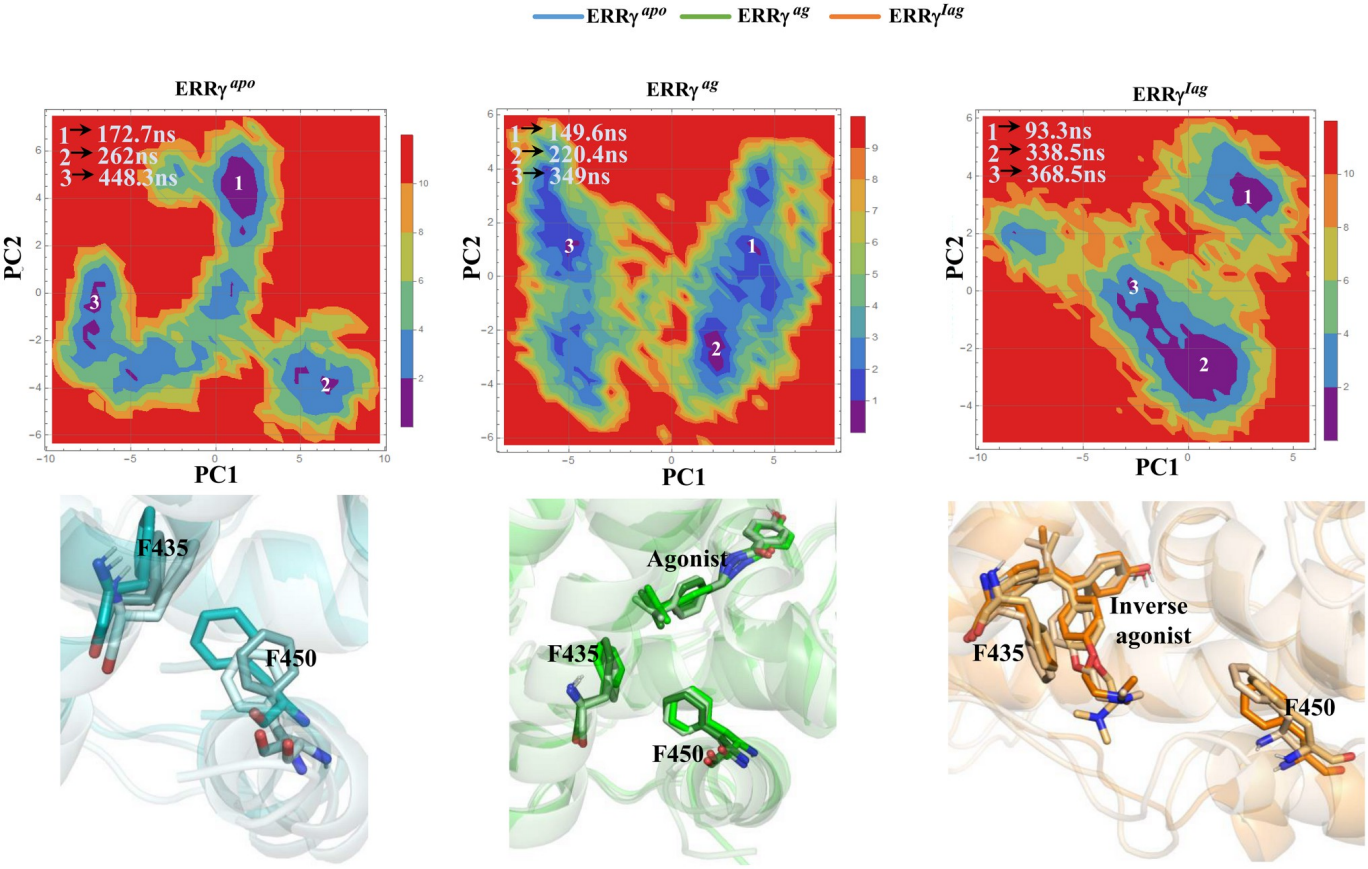
Free energy landscape of ERRγ^apo^, ERRγ^ag^ and ERRγ^iag^: 2D projection of free energy landscapes of ERRγ^apo^, ERRγ^ag^ and ERRγ^iag^ as a function of PC1 and PC2. The lowest point of the free energy reservoir shown in blue and violet represents the energy minima. The representative structures from each cluster of FEL were extracted and superimposed to show the variations in the AF-2 helix region. Residues F435, F450 and ligands are represented in the stick model.

Furthermore, we determined the global minimum energy conformation using free-energy landscape (FEL) analysis. The FEL map was derived by plotting PC1 and PC2 against Gibbs free energy. An FEL analysis of ERRγ^apo^ is shown in Fig 7. The plot shows a large distribution of conformations in the apo form with three minimal-energy clusters. On the other hand, when ERRγ^ag^ and ERRγ^iag^ were analyzed, the distribution of conformations was smaller than those of the apo form. ERRγ^ag^ showed two minimal energy clusters, whereas ERRγ^iag^ exhibited three strong clusters of minimum energy, with most of the structural conformations concentrated around the minimal cluster (Fig 7). This might be the reason for the reduced fluctuation of residues and the smaller distribution in the principal component analysis of ERRγ^iag^. In addition, when we analyzed the conformations of the F435 and F450 residues, we observed a large difference in conformations between ERRγ^ag^ and ERRγ^iag^. In Fig 7, F435 and F450 were aligned towards each other in ERRγ^apo^ and ERRγ^ag^, whereas in ERRγ^iag^, the inverse agonist positioned itself near F435 and deflected the F450 residue by steric hindrance. This steric hindrance caused the AF-2 helix to be displaced in the ERRγ^iag^ compared to the ERRγ^apo^ form.

### Betweenness centrality (C_B_)

To investigate the key residues required for the protein function, we performed network centrality calculations [49]. Centrality calculations can be successfully applied to identify specific residues present in the active site or co-factor binding site, along with allosteric networks in proteins [46]. In this study, we employed low-energy structures to construct residue interaction networks and calculated the betweenness centrality (C_B_). The residues (nodes) with high C_B_ values may have functional relationships and exhibit allosteric and protein-protein/ligand interactions [50].

C_B_ value plots for residues in ERRγ^apo^, ERRγ^ag^, and ERRγ^iag^ are shown in S1 Fig.

Residues with C_B_ ≥ 0.5, constituting ~10% of the C_B_ distribution, were selected for ERRγ^apo^, ERRγ^ag^, and ERRγ^iag^ structures. The residues are shown in Table 4, where ERRγ^apo^ had 16 residues, whereas ERRγ^ag^ and ERRγ^iag^ had 25 and 13 residues, respectively. Among them, few residues involved in hydrophobic interactions with the ligands (M306,V313 and V325 for agonist and L276, L309, V313 and L345 for inverse agonist). Moreover, V313 and L345 were previously reported to be involving in ligand interactions in ERRγ [51, 52].

**Table 4:** Critical residues in ERRγ^apo^, ERRγ^ag^, and ERRγ^iag^ with betweenness centrality values C_B_ ≥ 0.5. Residues involved in hydrophobic interactions are indicated in bold.

C_B_^apo^ ≥ 0.5	C_B_^ag^ ≥ 0.5	C_B_^iag^ ≥ 0.5
A251, Q302, A304, E307, I308, V313, V325, L361, K370, A373, L399, S428, V432, F450, M453 and L454	L241, A251, L271, D273, A283, F289, Q302, A304, W305, **M306**, E307, I308, **V313**, **V325**, A340, M359, L361, K370, I372, L391, L395, R413, V432, F450 and M453	**L276**, E307, **L309**, L311, **V313**, V314, **L345**, L352, K370, I372, L374, L395 and S428

**Fig 8:**
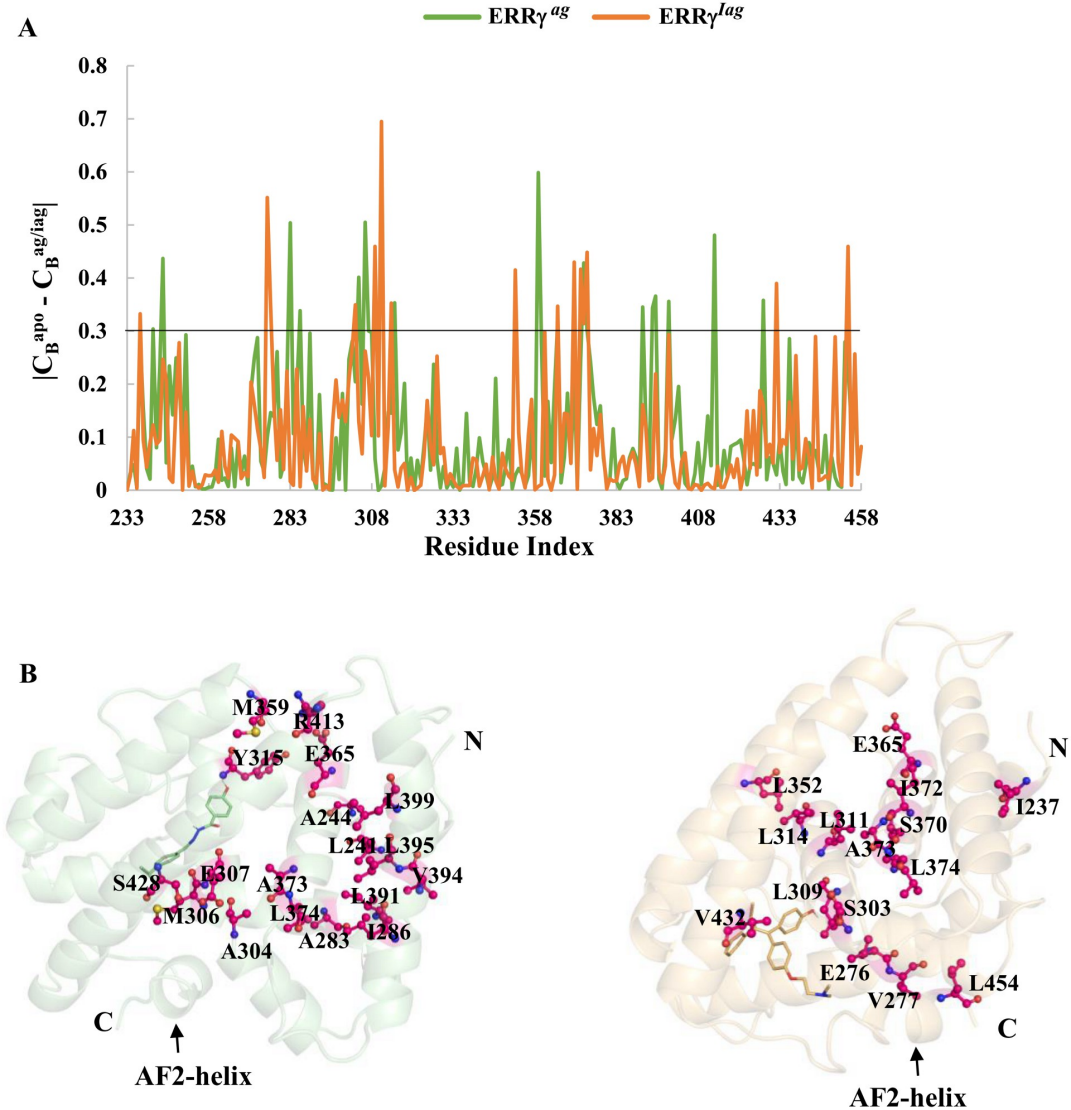
Network centrality analysis of ERRγ^apo^, ERRγ^ag^ and ERRγ^iag^ systems: **(A)** Plot showing the difference in C_B_ values between ERRγ^apo^ and ERRγ^ag^ / ERRγ^iag^. The threshold for identification of the residues |C_B_^apo^—C_B_^ag/iag^| was maintained greater than or equal to 0.3 (black line). **(B)** Critical residues identified by |C_B_^apo^—C_B_^ag/iag^| ≥ 0.3 are represented in hot pink using ball and stick model. Agonist GSK4716 and inverse agonist 4-OHT are represented in the stick model.

Further, we computed the difference in the C_B_ values (C_Bd_) of ERRγ^apo^ with ERRγ^ag^ and ERRγ^apo^ with ERRγ^iag^, and these residues were deemed to be critical if the C_Bd_ were larger (absolute value). The plot (Fig 8A) shows the C_Bd_ values of the residues in ERRγ^ag^ and ERRγ^iag^ with |C_B_^apo^—C_B_^ag/iag^| ≥ 0.3. From this result, we observed 18 residues for agonist and 15 residues for inverse agonist, respectively, which are shown in Table 5. Among these residues, M306 (for agonist), L276 and L309 (for inverse agonist) were involved in hydrophobic interactions with the ligands and few other residues located in the vicinity of binding pocket (Fig 8B). These results suggest that these residues might play an important role in ligand binding as well as protein function. Though these residues are suggested to be crucial, further experimental investigation would be required to unravel the functional and mechanistic importance.

**Table 5:** Critical residues with betweenness centrality values |CBapo—CBag/iag| ≥ 0.3. Bold residues represent residues common in both ERRγ^ag^ and ERRγ^iag^.

|C_B_^apo^—C_B_^ag^| ≥ 0.3	|C_B_^apo^—C_B_^iag^| ≥ 0.3
L241, A244, A283, I286, A304, **M306**, E307, Y315, M359, E365, A373, L374, L391, V394, L395, L399, R413 and S428	I237, **L276**, V277, S303, **L309**, L311, V314, Q352, E365, K370, I372, A373, L374, V432 and L454

## Discussion

The current study was conducted to identify the dynamics of ERRγ^apo^, ERRγ^ag^, and ERRγ^iag^, and the key residues responsible for the interactions between ERRγ and its agonist or inverse agonist. The structures retrieved from the PDB database served as the initial points of study and were simulated for 500 ns each. The first phase of this study was to determine whether the retrieved structures displayed reduced fluctuations in the simulation trajectory during the simulation period. RMSD studies of the protein structure and ligands showed that all three models exhibited conformational stability by reducing fluctuations throughout the simulation period. The Rg and SASA values also corroborated the RMSD results that the apo and bound forms of ERRγ were compact-folded during the simulation period. ERRγ^apo^ remained in the canonical three-layered α-helical sandwich throughout the simulation as reported earlier for other nuclear receptors [16]. The ligands 4-OHT and GSK4716 were anchored in the active site of the ERRγ structure throughout the simulation period. Previous studies have shown that the 4-OHT inverse agonist binds to pocket 1 of ERRγ, while the GSK4716 agonist binds to the larger pocket 2 of ERRγ [16]. Similar to the previous report, we observed that the ligands were positioned comfortably in the respective pockets. It is worthwhile to note that the pockets are adjacent to each other as shown in Fig 1.

Further, decreased residue flexibility among the ligand bound systems compared to apo ERRγ indicated that ligand binding stabilizes the protein by residue interactions (Fig 3). When the low energy structures were retrieved and analyzed for interactions, GSK4716 interacted with L268, L309, V313, R316 and Y326 of ERRγ. These interactions were also recorded earlier in a study involving bisphenol A with ERRγ, and they were found to be highly energetically favorable interactions [51]. On the other hand, in ERRγ^iag^, the hydrophobic interactions (with L268, C269, L309, V313, Y326, and L345) were similar to the interactions recorded for the agonists (Figs 4 and 5) [51]. The H-bonds observed for both agonist and inverse agonist, also suggested that, collectively, the binding of GSK4716 to ERRγ rigidified the entire protein and formed H-bonds with key residues of the protein (Fig 4). This is similar to a previous study where GSK4716 was identified as an agonist that was 50 times selective towards ERRγ [17]. In contrast, the inverse agonist 4-OHT formed fewer H-bonds (4-OHT forms H-bond with E275, however, less occupancy during simulations (Fig 4C)) with the residues in pocket 1. Moreover, the fewer hydrogen bonds, observed for the inverse agonist, suggested that hydrophobic interactions were crucial for the activity of the inverse agonist when compared to the agonist.

The PCA further showed the large dominant motions of apo ERRγ compared to ligand bound forms particularly on the loop regions (loop between helix 8 and 9) (Fig 6), which in turn reflected on the FEL with large metastable states for apo ERRγ. From RMSF studies, the larger fluctuations observed for the ERRγ^iag^, compared to ERRγ^ag^ in the region of the AF-2 helix showed that the inverse agonist binding displaces the AF-2 helix conformation [16]. The reason is that the binding of 4-OHT, which is a large molecule in a smaller ligand-binding pocket (280 Å^3^), causes displacement of the AF-2 helix. When snapshots from the minimum energy clusters of FEL were observed for ERRγ^ag^, both F435 and F450 aligned comfortably with few conformational changes. In contrast, the inverse agonist was positioned near the F435 residue, thereby deflecting the F450 residue. A previous study has shown that F435 is a crucial residue that prevents the common steroidal estrogens from binding to ERRγ and F450 displacement affects the volume available for ligand binding [16]. This steric hindrance between F435 and F450 caused the AF2-helix to be displaced, thereby, forcing it to adopt a conformation different from ERRγ^ago^ (Fig 7). The large residue fluctuations observed (Fig 3) in the AF-2 helix and end of helix 10 of ERRγ^iag^ were due to the presence of pocket 1 in the vicinity. As discussed earlier, the AF-2 helix is important for co-activator binding, and binding of 4-OHT changes the conformation of the AF-2 helix. This conformational change blocks the binding of any NR-box co-activator peptides and represses the activity of ERRγ [16, 53, 54]. The observed betweenness centrality (C_B_) of the residues and the difference in the betweenness centrality values (C_Bd_) further identified residues that were critical for intra-residue signal transduction and functioning of ERRγ [16, 51]. Few of the residues observed in C_B_ and C_Bd_ (Tables 4 and 5), were involved in hydrophobic interactions as well as in the vicinity of ligand binding pocket (Fig 8). Hence, we assume these residues might play a crucial role in ligand binding as well as in the functional activity of the protein.

## Conclusions

This study provides insight into the dynamic behavior of apo and ligand bound LBD of ERRγ and shed light into the residues involved in the binding of ligands to ERRγ. The results suggest the crucial residues for the interaction and functioning of ERRγ when bound to ligands. In addition, we observed that the conformation of the AF-2 helix upon binding to 4-OHT was maintained during simulations, and the residues F435 and F450 were involved in the conformational dynamics. Apart from agonist/inverse agonist studied here, several other compounds suggested in other studies may be capable of exhibiting similar effects. The major limitation of the study is the use of the only ligand bound LBD domain complex of the ERRγ. We assume that long-range molecular dynamic simulations on the ligand and coactivator bound LBD complex of ERRγ might provide a better understanding of conformational changes or allostery of ERRγ. Concluding from the results, we believe that the pharmacophore screening of ligands based on the results of this study may assist in identifying better drugs against ERRγ related diseases. Understanding the ERRγ dynamics as well as the binding of ligands to active pockets may also assist researchers design better therapeutic compounds for ERRγ.

## Supporting information

S1 Fig C_B_ values of ERRγ^apo^, ERRγ^ag^, and ERRγ^iag^ residues. (DOCX)
