# Supplementary figures and images for: Molecular dynamics of the ERRγ ligand-binding domain bound with agonist and inverse agonist
Source: PLoS One. 2023 Apr 6;18(4):e0283364. doi: 10.1371/journal.pone.0283364 (PMC10079097; doi:10.1371/journal.pone.0283364)

**Supplementary data**


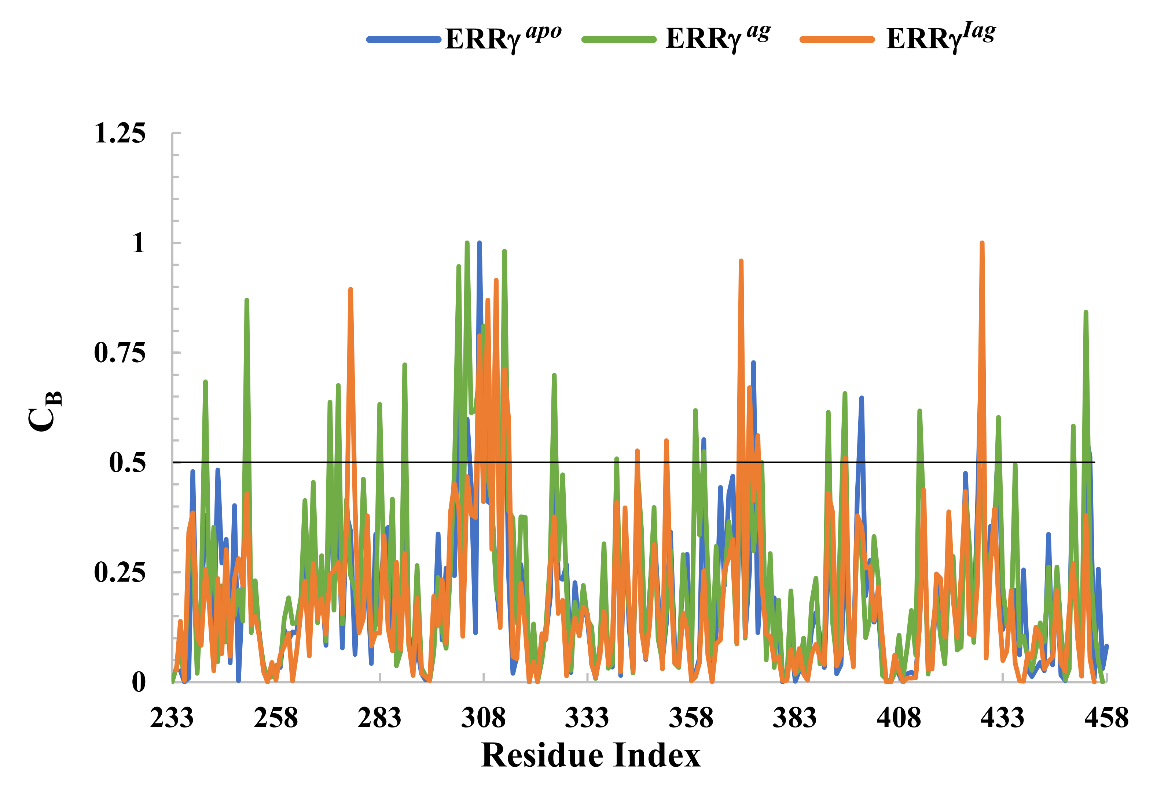


**Figure S1:** CB values of ERRγapo, ERRγag, and ERRγiag residues.

Supplement: S1 Fig — (DOCX) [file pone.0283364.s001.docx]
